# Using Entropy to Evaluate the Impact of Monetary Policy Shocks on Financial Networks

**DOI:** 10.3390/e23111465

**Published:** 2021-11-06

**Authors:** Petre Caraiani, Alexandru Vasile Lazarec

**Affiliations:** 1 Institute for Economic Forecasting, Romanian Academy, 050711 Bucharest, Romania; petre.caraiani@fabiz.ase.ro; 2 Faculty of Business Administration in Foreign Languages, Bucharest University of Economic Studies, 010374 Bucharest, Romania; 3 Department of Economics, Sociology and Law, The School of Advanced Studies of the Romanian Academy, 010071 Bucharest, Romania

**Keywords:** entropy, financial markets, monetary policy, networks

## Abstract

We analyze the changes in the financial network built using the Dow Jones Industrial Average components following monetary policy shocks. Monetary policy shocks are measured through unexpected changes in the federal funds rate in the United States. We determine the changes in the financial networks using singular value decomposition entropy and von Neumann entropy. The results indicate that unexpected positive shocks in monetary policy shocks lead to lower entropy. The results are robust to varying the window size used to construct financial networks, though they also depend on the type of entropy used.

## 1. Introduction

With the rapid increase of the use of network based approaches in economics, more and more key questions are approached from this perspective. The applications are diverse, and many times, they bring new insights. Without trying to exhaust an already large literature, we can mention the applications to business cycles [1], systemic risk [2,3,4,5,6], and contagion and spillovers [7,8,9].

In this paper, we aim at studying a less discussed topic from a network perspective. We aim at analyzing the transmission of monetary policy shocks to the financial markets. In this sense, we look at the way financial networks modify following monetary policy shocks. To measure the change, we use an entropy measure of networks based on singular value decomposition and on von Neumann entropy. The main question of this paper is as follows: do monetary policy shocks impact the financial networks?

There are several directions in which we contribute to the literature. Our first contribution is related to the analysis of monetary policy shocks in the context of networks. There is a rapidly growing literature, with contributions mainly focusing on the role of production networks. A reference paper in this direction is one published by Weber and Ozdagli [10], who used the spatial structure of production in the United States (based on the input–output structure) to measure how this affects the transmission of monetary policy shocks. We can also mention Caraiani et al. [11], who studied the propagation of monetary policy shocks using specific measures of production networks such as upstreamness and downstreamness, finding that they matter for the transmission of monetary policy shocks. In a related paper [12], Caraiani studied in an international context the transmission of oil shocks using network measures such as density and skewness of links, finding again that the network structure matters significantly for the transmission of (oil) shocks.

A second contribution is to the field of financial networks. We have already cited some reference papers applying networks approaches to the financial markets. Here, we contribute to the understanding of the relationship between monetary policy shocks and the stock market, but we also quantify the structural changes in the financial markets networks using an entropy measure.

Finally, our last contribution is related to the use of entropy in measuring the changes in the financial markets networks following a particular shock—here, a monetary policy shock. There are various ways to measure the entropy of financial markets. A discussion of the various ways to measure entropy in financial networks is conducted in [13]. Below, we also review a few contributions on this topic and outline our contribution in this direction.

A significant contribution was made by [14], who applied transfer entropy to study the relationships between 187 companies in the world. This approach allowed him to identify a central role for insurance companies from large economies such as the United States and the Euro Area.

A different contribution looked at the ability of singular-value-based entropy of financial markets to signal the state of the market. In [15], entropy is measured using a singular value decomposition of the matrix of correlations of financial stock. Caraiani also showed that this entropy measure has predictive ability for the dynamics of the stock market. More recently, Caraiani [16] extended the work to the case of international financial markets, showing that there are spillovers of entropy between the big financial markets, with entropy measured again based on the singular value based decomposition of the financial stock.

Another contribution on the use of entropy was given by Bekiros et al. [17], who used entropy measures of the financial markets to show that there is a decoupling between commodity and equity markets. Another approach consisted of showing that we can use permutation entropy to analyze how the degree of information changes during a market crash [18]. The main result of this latter paper was that during financial market crashes, the permutation entropy decreases.

In this paper, we extend previous research from several literature strands (i.e., networks and monetary policy, financial networks, and entropy of financial networks) by making several contributions. First, we show that we can approach the topic of the changes in the financial networks following monetary policy shocks using networks measures. As far as we know, this has not been studied extensively before. There are, however, a few studies that are close to ours. For example, Beltran et al. [19] found that Fed Funds networks (along with abundant reserves) tend to dampen the impact of monetary policy transmission. In a different framework, using agent-based modeling, Riccetti et al. [20] found a significant role for a financial accelerator that was founded on three dimensions: a leverage one, a stock market one, and a network one. We can also mention the study by Silva et al. [21], who consider a granular approach that takes into consideration the network relationships between agents, along with the balance sheets compositions. The inclusion of network data allowed them to study contagion effects as well. They applied their model to Brazilian data. However, our focus is rather on detecting the changes in financial networks following monetary policy shocks.

Second, we quantify the changes in the financial markets networks based on different measures of entropy. Different from previous studies, we focus on event studies with monetary policy shocks precisely identified following FED communications. Thus, we can to isolate the impact of such announcements by considering the changes in entropy. Since we consider windows of data before and after these announcements, we can isolate the impact solely of these announcements.

The paper is structured as follows. We first discuss the methodology used throughout the paper in the following section. In the third Section, we present the data used in the empirical analysis. In the fourth Section, we perform the empirical analysis by looking at the impact of monetary policy shocks on financial networks. Finally, in Section five, we discuss the results and suggests possible extensions of the present result.

## 2. Methodology

We detail here the main tools used in the empirical analysis. While most of the methods applied here have been extensively used in the literature, they might still be unknown to some readers.

Our research builds on the recent work on financial networks and measuring the key properties of networks by applying network-based measures of entropy. We use these well-studied and understood methods to approach the impact of monetary policy shocks on financial networks in a novel manner.

### 2.1. Correlation Networks of Stocks

In the first stage, we construct correlation-based networks. Since our sample of financial series comprises the components of the Dow Jones Industrial Average index (while considering also the historical changes in its composition; see Appendix A for the composition of the DJIA index), we construct time-varying correlation matrices of the returns. Returns for a stock *i*, $r_{i}$, are measured through the log-difference in prices at time *t*, i.e., $P_{i}\left(t\right)$, namely:

(1) $$r_{i}\left(t\right)=log\left[P_{i}\left(t\right)\right]-log\left[P_{i}\left(t-1\right)\right]$$

We further compute the standard correlation between two stocks using:

(2) $$\rho_{i,j}=\frac{cov(r_{i},r_{j})}{\sigma_{r_{i}}\sigma_{r_{j}}}$$

Here, $r_{i}$ represents again the return of the stock *i*. $\sigma_{r_{i}}$ is the standard deviation for the return of the stock *i*.

Our approach to constructing networks relies on correlations. While we admit that there are other approaches, this approach remains one of the standard ones. Further discussions on this can be found in [22], in which the authors discuss various approaches based on correlations, and also in [23], in which the authors use VAR models and compute the financial networks based on the variance decomposition.

Once correlation matrices are obtained, we can derive the corresponding adjacency matrix and build the financial networks. Our focus is on measuring the structural change in financial networks (and their corresponding adjacency matrices) following changes in monetary policy, as revealed by the series in monetary policy announcements (see below).

### 2.2. Singular-Value-Decomposition-Based Entropy

Many measures can be used to characterize a (financial) network quantitatively. Here, we focus on a simple measure that measures the degree of entropy in a (financial network). This measure has been used in the past, and it was shown to have significant predictive and/or informational content for a financial network (see [15,16]).

We start from a standard singular value decomposition (SVD, hereafter) applied to the adjacency matrix that we have obtained:

(3) $$A=USV^{T}$$

The SVD decomposition is applied to the adjacency matrix of returns as computed in Section 2.1, denoted by A, characterized by *n* rows and *n* columns (the matrix is square). Furthermore, the resulting matrix *U* has *n* rows and *n* columns, and the matrix *V* has *n* rows and *n* columns. We can further write the matrix *S* as follows:

(4) $$S=\mathrm{diag}(\lambda_{1},\lambda_{2},\ldots ,\lambda_{n})$$

The literature shows that the resulting matrix consists only of positive elements, which are also ordered decreasingly.

We further employ the SVD decomposition to measure the entropy of the adjacency matrix and, implicitly, of the financial networks that we built. There are many ways to measure the entropy of a financial network and market; for examples, see the recent contributions by Nie and Song [13] and Anand and Bianconi [24]. The approach employed here is based on the key contribution by [25] as well as the more recent application by [26].

To measure the entropy, we use the approach employed in [15,26,27] in the context of financial networks. Based on the singular value derived above, $\lambda_{n}$, we first compute the normalized values using:

(5) $${\bar{\lambda }}_{k}=\frac{\lambda_{k}}{\sum \lambda_{k}}$$

In the next step, we measure the entropy employing the normalized singular values as follows:

(6) $$En=-\sum {\bar{\lambda }}_{k}ln\left({\bar{\lambda }}_{k}\right)$$

Our key measure of interest is En, which stands for the singular-value-decomposition-based entropy. We can measure the entropy for each financial network that we construct. As explained in the empirical part, we will aim to measure the changes in the financial networks following monetary policy shocks. Details about constructing the change in the entropy are given in Section 4.1.

### 2.3. Von Neumann Entropy

To obtain the von Neumann entropy, denoted by $En_{vne}$, we use the Laplacian matrix of the graph, denoted by *L*. If Λ is the spectrum (or the set of eigenvalues) of the Laplacian matrix, we can compute the von Neumann entropy via a similar formula:

(7) $$En_{vne}=-\sum_{\lambda \in \Lambda }\lambda_{k}ln\left(\lambda_{k}\right)$$

We also consider the normalized Laplacian, $\bar{L}$ for which we compute the set of eigenvalues $\bar{\Lambda }$, and derive a normalized measure of entropy (denoted by $En_{vnen}$):

(8) $$En_{vnen}=-\sum_{\lambda \in \bar{\Lambda }}{\bar{\lambda }}_{k}ln\left({\bar{\lambda }}_{k}\right)$$

## 3. Data

We selected data for the stock components of the Dow Jones Industrial Average Index, DOW30 (see Appendix A). We selected daily data to ensure larger samples and thus to construct financial networks before and after monetary policy announcements. Contrary to previous studies (see, for example, [15]), we also account for the time changes in the Dow Jones structure since the index is updated constantly. Given the components’ historical changes, we update the series used following the official date when changes occurred. To simplify the analysis, we focus only on data starting with 2000.

**Figure A1:**
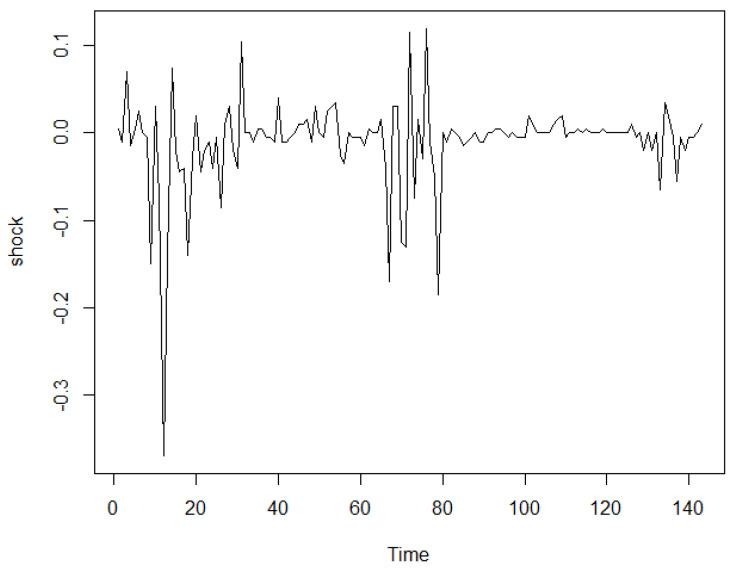
Monetary policy shocks.

A second series we use is that of monetary policy announcements. This is based on the study by [28]. We then use the updated data set of announcements as in [29]. The data set spans from 1990 to 2016, but we focus only on 2000 to 2016. Appendix B, Figure A1 displays the monetary policy shocks series. The time *x*-axis indicates the observations that we have on the dates shocks produce. Shocks are produced at irregular dates. The *y*-axis stands for the magnitude of the shock that is observed.

## 4. Results

### 4.1. Measuring the Entropy

In this section, we derive the measure of entropy that we are interested in. In contrast to previous contributions that have also used singular-value-based entropy to characterize financial networks (see [15] or [16]), we focus here on measuring the change in the singular-value-based or von Neumann entropy in pre- versus post- dates when the monetary policy shocks occur.

**Figure 1:**
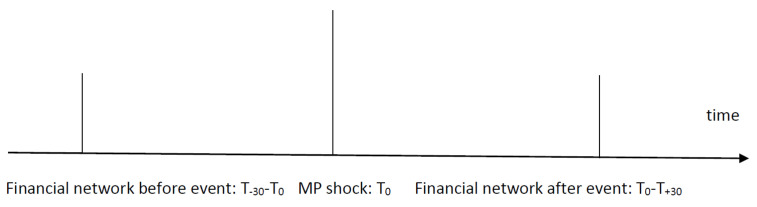
The informational timing. Note: $T_{0}$ is the moment the monetary policy is produced, $T_{-30}-T_{0}$ is the window of 30 days before the event, and $T_{0}-T_{+30}$ is the window after the monetary policy shock. The size of the window is varied for robustness reasons.

An informational issue is also critical to ensure that the change in the financial network(s) comes only from the current monetary policy shock. To counter this effect, we isolate the impact of each monetary policy shock by considering the state of the financial network (as characterized by the entropy) before and after each event (or monetary policy shock). The informational issue that we discuss is represented below, see Figure 1:

While previous studies (see [15,16]) relied on a sliding window, here, we compute for each monetary policy shock that entropy for the financial networks before the event and after the event. We consider a window for which the financial network is derived. This is used only before and after the event. Thus, the resulting series is the series of changes in the singular-value-based entropy ex post compared to ex ante.

To control for the robustness of the results to the size of the window used, we vary the window’s dimension and consider windows of 20, 30, and 45 days (these correspond to calendar days, while the trading days are just at most five each week). Our main interest relies on financial networks that are affected only by the events we consider, i.e., monetary policy shocks. In this sense, considering that only windows of 20, 30, or 45 days fulfills this essential criterion while ensuring enough observations to construct the financial networks. We also consider a larger window of 60 days (with the results available at request) for robustness. However, it might sometimes overlap with previous or subsequent monetary policy shocks, but this should be taken only as additional evidence.

In Appendix B and Appendix C, we show the log-difference of the Shannon entropy measure for the different window sizes used, namely for 20, 30, and 45 days (the figures for von Neumann entropy are similar, and they are available at request). Our data start from 2000, ensuring that there are enough observations to carry the statistical analysis. The dot-com crisis from 2001 is marked through a decreasing value of entropy. A similar pattern is noticed after about eight years, corresponding to the timing of the great financial crisis. The *x*-axis is interpreted as showing the observations on entropy changes, while the *y*-axis shows the magnitude of the change.

### 4.2. The Impact of Monetary Policy Shocks on Financial Networks

This section aims to answer the paper’s central research question: do monetary policy shocks impact the financial networks? We use the singular-value-based entropy and von Neumann entropy measures derived in the last section to answer this question. We test the hypothesis of whether monetary policy shocks have a significant impact on financial networks as measured through the change in the entropy of the financial networks.

We consider the following basic regression models. The model aims at capturing the relations between the shocks (the change in the monetary policy stance) and the entropy of the financial markets.

(9) $$dEn_{t}=c+MP_{t}+u_{t}$$

Here, $dEn_{t}$ is the change in the entropy, while $MP_{t}$ are the monetary policy shocks. *c* is a constant, while $u_{t}$ are the residuals of the regression. For robustness, we use the Shannon entropy and the von Neumann entropy, including one based on a normalized version of the Laplacian.

Before performing the regression, we also test for the unit root in both monetary policy shocks and the change in entropy for various window sizes. The results are shown in Appendix D. The unit root hypothesis is strongly rejected in each case, for either of the monetary policy shocks, for the different measures of entropy based on different approaches and window sizes.

Table 1 shows the regression results described in Equation (1) for the Shannon entropy for the various window sizes considered: 20, 30, and 45 days. Although the $R^{2}$ is low, the *F*-test indicates that the model is significant from a statistical point of view (For those not familiar with the regression analysis, the *F*-test is an overall test of significance for the estimated regression. The null hypothesis is that the model does not have significant explanatory power).

However, the key result is the statistically significant and negative coefficient associated with the MP shock in each case considered. In other words, monetary policy shocks lead to a reduction in the financial network’s entropy, as measured by the change in the singular-value-based entropy. The results are robust to the window size used, and they tend to become stronger for larger windows.

Additionally, we consider in Table 2 and Table 3 the von Neumann entropy, varying the window size as well. However, the results are not statistically significant.

In Appendix E, we further test whether controlling for the correlation threshold changes the results for the Shannon entropy (we set the correlation weaker than 0.30 to 0). We were able to derive the results only for two types of windows of 20 and 30 observations. The results remain negative and statistically significant, and the magnitude is even larger than for the baseline case. We tried the same exercise for the von Neumann entropy; however, the results remained the same.

## 5. Discussion

In this paper, we aimed at approaching the issue of monetary policy effects on the financial markets from a network perspective. We analyzed whether monetary policy shocks statistically impact the financial networks (as constructed from the Dow Jones Industrial Average components). To measure the change in the financial networks, we used the change in the entropy (either singular-value-based or von Neumann).

The main contribution of this paper was to show that monetary policy shocks have indeed a statistically significant impact on financial networks: a positive monetary policy shock (corresponding to a tightening of the monetary policy and a higher interest rate) had a negative impact on the singular-value-based entropy of the financial networks. Our results are robust to varying the size of the window used to construct the financial networks. They are also robust to controlling for the significance of correlation. However, the results using the von Neumann entropy are not statistically significant.

The interpretation of the result is that the release of the new information through the Fed communications on the interest decreases the entropy of the financial market networks. This is a somewhat expected result since it reduces the degree of uncertainty in the financial markets.

There are a few novel results that can be outlined. First, we highlight the fact that monetary policy shocks do affect the financial networks. Previous studies (see [10,12,16]) considered (production) networks that are invariant to changes in aggregate shocks, including monetary policy shocks. Our focus was on financial networks and how they respond to monetary policy shocks. Second, we also show that entropy measures of networks can be used to detect the changes in financial networks. This has been used before in a few studies; however, in this paper, we show that event studies can be combined with entropy to evaluate the impact of financial networks.

The results here can be further extended in various ways. For example, one can consider different ways to construct entropy from financial networks. Furthermore, financial networks can also be characterized in many ways, including based on measures that are more intuitively linked to financial and economic concepts (such as risk, for example), which can be further used to analyze the impact of monetary policy shocks in a network context.

## Figures and Tables

**Table 1:** Monetary policy shocks and the change in Shannon entropy for different window sizes.

Variable	Entropy +/− 20 Days	Entropy +/− 30 Days	Entropy +/− 45 Days
Intercept	−0.06351 **	−0.05160	−0.01865
MP shock	−1.28732 **	−1.58216 **	−1.55791 **
$R^{2}$	0.04149	0.04576	0.04374
*F*-test	6.06 **	6.714 **	6.403 **

Note: * denotes statistical significance of the *F*-test at the 0.10 level, ** statistical significance at the 0.05 level, and *** at the 0.01 level.

**Table 2:** Monetary policy shocks and the change in von Neumman entropy for different window sizes: normalized.

Variable	Entropy +/− 20 Days	Entropy +/− 30 Days	Entropy +/− 45 Days
Intercept	0.006243	0.001215	−0.0005439
MP shock	0.106932	0.070585	0.0608659
$R^{2}$	0.01106	0.007153	0.008522
*F*-test	1.566	1.009	1.203

Note: * denotes statistical significance of the *F*-test at the 0.10 level, ** statistical significance at the 0.05 level, and *** at the 0.01 level.

**Table 3:** Monetary policy shocks and the change in von Neumman entropy for different window sizes: not normalized.

Variable	Entropy +/− 20 Days	Entropy +/− 30 Days	Entropy +/− 45 Days
Intercept	0.006880	0.001546	0.000870
MP shock	0.135886	0.089820	0.089003
$R^{2}$	0.01425	0.01599	0.01261
*F*-test	2.024	2.016	1.788

Note: * denotes statistical significance of the *F*-test at the 0.10 level; ** statistical significance at the 0.05 level, and *** at the 0.01 level.

**Figure A2:**
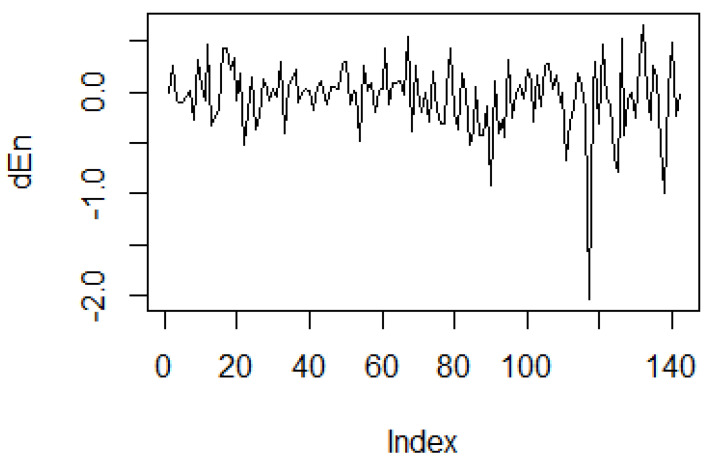
The change in singular-value-based entropy-20 days window.

**Figure A3:**
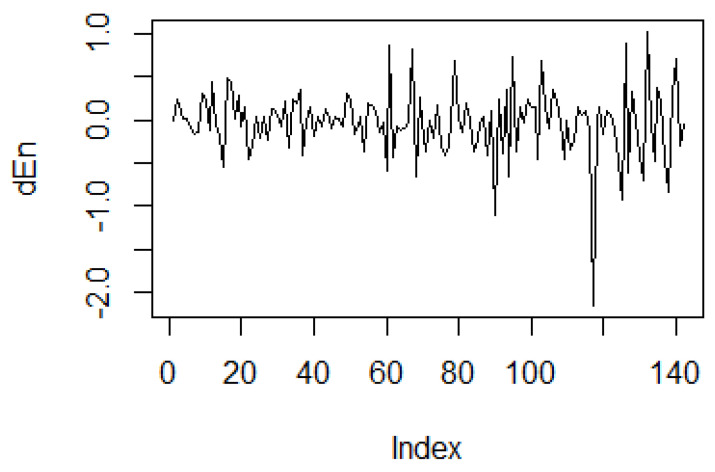
The change in singular-value-based entropy-30 days window.

**Figure A4:**
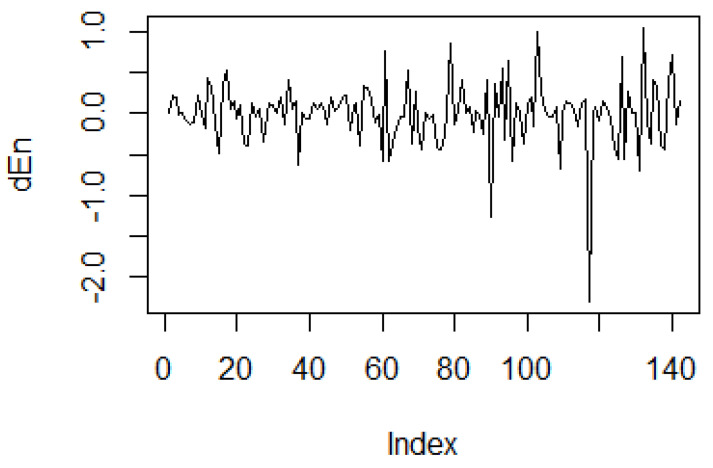
The change in singular-value-based entropy-45 days window.

## Abbreviations

The following abbreviations are used in this manuscript:

DJIA	Dow Jones Industrial Average
SvdEn	Singular Value Decomposition Entropy
MP	Monetary Policy
ADF	Augmented Dickey–Fuller
PP	Phillips–Perron

## Appendix A. DJIA Index Components

**Table A1:** DOW Jones Industrial Average Components as of end of 2015.

Company	Abbreviation
3M Company	MMM
American Express Company	AXP
Apple Inc.	AAPL
Boeing Company	BA
Caterpillar, Inc.	CAT
Cisco Systems, Inc.	CSCO
Chevron Corporation	CVX
Dow Chemical Company	DD
Exxon Mobil Corporation	XOM
General Electric Company	GE
The Goldman Sachs Group, Inc.	GS
Home Depot, Inc. (The)	HD
Intel Corporation	INTC
International Business Machines	IBM
Johnson & Johnson	JNJ
JP Morgan Chase & Co.	JPM
Coca-Cola Company (The)	KO
McDonald’s Corporation	MCD
Merck & Company, Inc.	MRK
Microsoft Corporation	MSFT
Nike, Inc.	NKE
Pfizer, Inc.	PFE
Procter & Gamble Company (The)	PG
Raytheon Technologies	RTX
The Travelers Companies, Inc.	TRV
United Health Group Inc.	UNH
Verizon Communications Inc.	VZ
Visa Inc.	V
WalMart Stores, Inc.	WMT
Walt Disney Company (The)	DIS

## Appendix D. Unit Root Tests

**Table A2:** Unit Root Tests: Shannon Entropy.

Country	ADF Test	PP Test
Monetary Policy Shock	−5.7318 **	−124.91 **
Entropy—20 days window	−4.8608 **	−136.29 **
Entropy—30 days window	−5.4853 **	−159.48 **
Entropy—45 days window	−5.7318 **	−162.22 **

Note: * denotes statistical significance of the unit root test at 0.10 level; ** at 0.05 level; *** at 0.01 level.

**Table A3:** Unit Root Tests: Von Neumann Entropy—normalized.

Country	ADF Test	PP Test
Entropy—20 days window	−4.7529 **	−121.16 **
Entropy—30 days window	−4.555 **	−186.99 **
Entropy—45 days window	−5.0434 **	−172.26 **

Note: * denotes statistical significance of the unit root test at the 0.10 level, ** at the 0.05 level, and *** at the 0.01 level.

**Table A4:** Unit Root Tests: Von Neumann Entropy—not normalized.

Country	ADF Test	PP Test
Entropy—20 days window	−4.8207 **	−116.98 **
Entropy—30 days window	−4.4355 **	−185.88 **
Entropy—45 days window	−5.0565 **	−170.02 **

Note: * denotes statistical significance of the unit root test at the 0.10 level, ** at the 0.05 level, and *** at the 0.01 level.

## Appendix E. Robustness Exercise: Taking into Account a Correlation Threshold

**Table A5:** Monetary policy shocks and the change in Shannon entropy for different window sizes. Controlling for correlation threshold.

Variable	Entropy +/− 20 Days	Entropy +/− 30 Days
Intercept	0.11335 ***	0.15085 ***
MP shock	−1.72342 **	−1.88651 ***
$R^{2}$	0.04601	0.0483
*F*-test	6.75 **	7.125 ***

Note: * denotes statistical significance of the *F*-test at the 0.10 level, ** statistical significance at the 0.05 level, and *** at the 0.01 level
